# Comparison of affective responses between an online and in-person high-intensity functional training session in sports students

**DOI:** 10.3389/fspor.2026.1916043

**Published:** 2026-09-24

**Authors:** Anine Brudeseth, Tom Erik Jorung Solstad, Amund Riiser, Atle Hole Sæterbakken, Espen Hermans, Jon Ingulf Medbø, Ove Sollie, Vidar Andersen

**Affiliations:** 1 Faculty of Education, Arts and Sports, Western Norway University of Applied Sciences, Sogndal, Norway; 2 Department of Physical Performance, Norwegian School of Sports Sciences, Oslo, Norway

**Keywords:** group training, high intensity interval training, in-person sessions, online training, physical active young adults

## Abstract

The aim of this study was to compare affective responses between a synchronous online and an in-person high-intensity functional training (HIFT) group exercise session on perceived effort, discomfort, displeasure/pleasure, enjoyment and preference in sports students. Using a randomized crossover design, 24 participants (14 men, 10 women, mean age 22) were randomized into two groups. Both groups completed in randomized order two standardized HIFT sessions, one in-person and one synchronous online. Rating of perceived effort, rate of perceived discomfort, session displeasure/pleasure and exercise enjoyment were measured immediately after ending each session. In addition, 48 h after the last session the participants answers which session they preferred and gave reasons for their preference. The reported pleasure (2.0 ± 2.2 vs. 3.1 ± 1.7, ES = 0.47, scale −5 to +5) and exercise enjoyment (4.2 ± 1.2 vs. 5.1 ± 1.0, ES = 0.56, scale 1–7) were lower conducting the online HIFT session than the in-person HIFT session. The online HIFT session were perceived less effortful than the in-person HIFT session (5.6 ± 1.7 vs. 6.7 ± 1.7, respectively, ES = 0.48, scale 0–10), but no difference in perceived discomfort (4.4 ± 2.0 vs. 4.7 ± 1.9, ES = 0.12, scale 0–10). There was no detectable cross-over effect of the order of the two training sessions (*p* ≥ 0.29). The large majority of participants (*n* = 22, 92%) preferred in-person group exercise, while only two participants (8%) preferred the online session. In sports students with prior group-training experience, in-person HIFT session demonstrated great perceived effort, pleasure and enjoyment when using self-reported perceptual measures, and was the preferred exercise modality compared with the online session.

## Introduction

During the Covid-19 pandemic, online training, such as live streaming workouts or prerecorded sessions, became the number one fitness trend worldwide (1). While initially a forced necessity, online training has since been established as a popular concept within the fitness industry driven by high accessibility and time efficiency (2, 3). Furthermore, online training may reduce barriers to entry and engage a broader populations, thereby, potentially positively impact public health (4).

The physiological demands of online training can match in-person sessions, as demonstrated by equivalent oxygen uptake and METs during online delivery (5) and comparable cardiorespiratory adaptations during high-intensity interval training (HIIT). In the last decade, there has been a growing interest in time-efficient modalities as HIIT and high-intensity functional training (HIFT) to improve muscle strength, aerobic power, and body composition across different populations (6, 7). Both HIIT and HIFT include high-intensity workouts with brief rest between sets and exercises. HIIT typically includes interval training and has proven efficient to improve cardiorespiratory fitness outcomes and reduce fat mass (8, 9). In contrast HIFT also includes resistance training and has proven efficient to improve muscle strength, power, and hypertrophy (6, 10). To achieve and sustain these high physiological demands in a home-based setting, HIFT emerge as a potential preferred modality as the format allows for high cardiovascular exertion with minimal requirements for space and equipment (11).

Traditionally, group-based HIFT is social by nature (12). However, this social component may be compromised in the transition to an online platform. Consequently, exercise enjoyment is reported as significantly lower during online training compared with traditional in-person attendance (13, 14). In online environments, intrinsic motivation can be diminished by the absence of non-verbal cues and reciprocal communication, limited audiovisual feedback from peers, and challenges in forming relationships (14, 15). Despite a growing body of literature comparing online and in-person training, there are several knowledge gaps that needs to be addressed. For example, the majority of the previous studies have compared responses conducting yoga or Zumba (5, 14) which may be considered as a more viewable online training approach than HIFT. Others have included a mixture of different fitness classes (13, 14) which makes it challenging to interpret the findings as a result of online training *per se*. Another limitation is the inclusion of only a few affective responses (13), and ignored outcomes like effort or discomfort. The population recruited has been middle-aged adults, elderly and individuals with obesity (13, 15, 16), whereas young adults has not been included. Finally, there is a lack of controlled, within-subject experimental designs comparing the same session conducted online and in-person. To summarize, affective responses and preference during an online vs. in-person HIFT exercise is unclear. More specifically, and to our best knowledge, no studies have quantified differences in perceived effort and affective responses after standardized high-intensity training protocol where the same healthy participants are exposed under both.

The aim of the present study was, therefore, to explore and compare affective responses of an online group HIFT session compared with an identical in-person session in sports students. Our primary outcome was how the different sessions affect the perception of session pleasure and enjoyment while our secondary outcome was the perception of effort and discomfort.

## Methods and materials

### Study design

The present study is a randomized crossover design comparing a synchronous online exercise session with an in-person group exercise session using questionnaires. The participants were randomized into two groups where both groups attended one in-person and one online exercise session but in opposite and randomized order. After each training modality, the participants reported rate of perceived effort (RPE), rate of perceived discomfort (RPD), session pleasure/displeasure feelings (sPDF), and scores on an exercise enjoyment scale (EES). Forty-eight hours after completing both sessions, the participants were asked to report their preferred training modality (online or in-person) and reasons for their preferences.

### Participants

Due to the exploratory approach, no power sample analysis was conducted before starting the project (17). Instead a sample of convenience, based on the available resources for this specific project, was used. A total of 24 participants (14 men, 10 women) with mean age 22 were recruited among sports students from the university. The participants were considered as physical active and free of injuries or pain that could limited maximal effort. The average training volume were 2 -3 sessions per week. Inclusion criteria were: (a) adults (18–30 years), (b) prior experience with group training, (c) a physical fitness level that enable them to conduct strength exercises (i.e., push-ups, sit-ups, squats) with their own body-weight, (d) access to a computer with videoconferencing software (Zoom, Zoom Video Communications, Inc., San Jose, CA, USA), (d) being injury-free, (e) physically capable of performing bodyweight exercise.

The study was approved by the Norwegian Centre for Research Data (NSD; reference number 609778) and conducted in accordance with institutional ethical guidelines. All participants were informed orally and in writing about the protocol and procedures and had to sign an informed consent to be enrolled in the study. Participation was voluntary, and participants could withdraw at any time without any consequences for the participant.

### Procedures

The participants were randomly assigned into two groups of 12 individuals. Both groups completed two standardized HIFT sessions (6). One conducted synchronously online via Zoom (Zoom Communications, Inc, San Jose, California, USA) and one conducted in-person at a training fitness facility. One group completed the online training session first followed by the in-person training session, whereas the other group completed the sessions in reverse order. The training sessions were separated by one week (Figure 1). All four sessions were led by the same certified instructor with 12 years of experience with teaching group based exercise-classes. The session content and delivery were standardized across the modalities, including identical exercises, music and instructional cues. During the online session, the instructor viewed all participants live through the computer screen, and the participants could also see each other. The only variable that differed between the training sessions was the mode of delivery (online or in-person).

**Figure 1:**
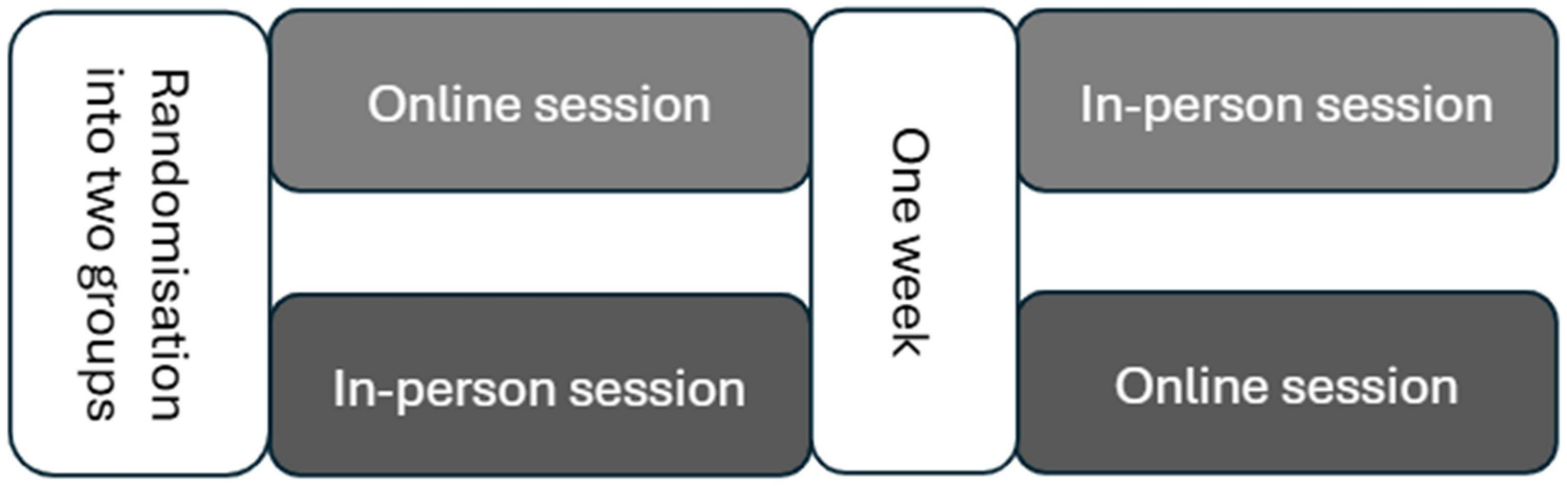
Study design.

Each session consisted of a 10-minute warm-up and a 30-minute HIFT session. The warm-up included a general warm-up with mobility exercises and a specific warm-up including two series with eight repetitions of the exercises in the HIFT session. The HIFT session included 12 exercises organized into six work period of 4 min followed by a 1-minute rest. Each work period consisted of two exercises performed for 30 s followed by 10 s of rest. This was repeated three times (Figure 2). All work periods were performed to pre-selected music with tempo (beats per minute, bpm) matched to the intended exercise intensity. The exercises performed in the work periods were jumping squats and push-ups (154 bpm), jumping lunges and mountain climbers (128 bpm), Skate jump and burpees (130 bpm), high knees and diagonal rise (159 bpm), jumping Jacks and diagonal stomach (or leg rise) (128 bpm), and Sit-ups and hip thrusts (125 bpm). The students were recommended to follow the instructor in terms of technique, tempo and execution of all exercises. The music was identical for both modalities. In the online session, the participants received the play list and played the music on their own stereo while the cues and instructions from the instructor were received through the video conference audio. All participants were instructed to turn on the camera, and the instructor used a monitor to see every subject during the online session.

**Figure 2:**
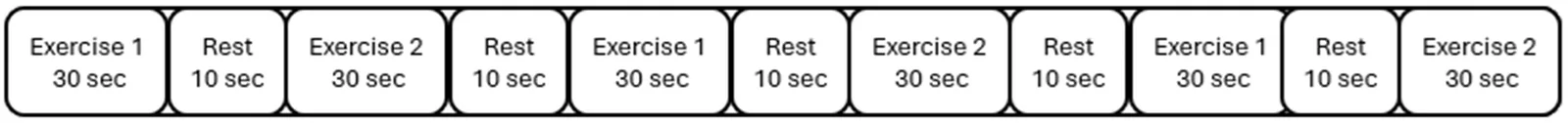
Illustration of one of six working periods.

### Measurements

Within five minutes after each training session the participants completed standardized sets of four questionnaires (16, 18, 19). The scales were translated from their original versions into Norwegian according to recommended protocol (20). Briefly, three independent researcher with advanced proficiency in English translated the scales, after which they compared their versions, discussed discrepancies, and reached agreement on the final translations. These finalized Norwegian versions were then back-translated into English by a professional translator. The back-translated English versions were subsequently compared with the original scales. Overall, only minor differences were identified, and these were resolved through mutual agreement.

The questionnaire assessed the rate of perceived effort (RPE, 0–10 scale), rate of perceived discomfort (RPD, 0–10 scale), session pleasure/displeasure feelings (sPDF, −5 to +5 scale), and exercise enjoyment scale (EES, 1–7 scale) (16, 18, 19). The scales were presented and explained in details to the participants (visually and orally) after each completed training session as previously recommended (21). The RPE and RPD scales were anchored as described in previous studies (22, 23) where 0 was consider as no effort and 10 was consider as maximal effort. The sPDF scale used a 11-point bipolar scale ranging from −5 (very bad) to 5 (very good) where 0 was consider neutral. The EES ranged from 1 (not at all) to 7 (extraordinary). The Norwegian version of the scales have previously been pilot tested and used in studies with a comparable population (22, 24). Of note, the Norwegian versions have, to the best of our knowledge, not been validated, however, the original versions have all been claimed to be valid and reliable (16, 19, 25).

Forty-eight hours after the last training session, the participants received a second questionnaire by e-mail. The participants were asked: Which of the two group training sessions (i*n-person group training in a studio or group training* via *Zoom with online participation*) do you prefer and further reasons for their preference. The participants returned the questionnaire via e-mail.

## Data analysis

### Quantitative data

Data are presented as mean with standard deviation. Statistical analyses and visualization of data were performed using JASP (0.97, University of Amsterdam, Amsterdam, The Netherland's). Difference in affective responses between training modalities was examined by Wilcoxon signed rank test. Effect size (ES) was calculated as the product-moment correlation, with the following equation: ES = *z*/√*n*, with *z* being the *z*-value from the Wilcoxon signed rank test and *n* being the number of participants. An ES of 0.10–0.29 was considered small, 0.30–0.49 medium, and ≥0.50 large (26). Spearman's correlation analysis examined the potential relationships between affective responses (RPE, RPD, EES and sPDF). The magnitude of the effect for the correlations was based on the following scale. Small: ≤0–0.30, moderate: 0.31–0.49, large: 0.50–0.69, very large: 0.70–0.89, and nearly perfect: ≥0.90 (27). A *p value* < 0.05 was taken as reflecting statistical significance. When single tests reported a *p*-value less than 0.05, a Bonferroni-Šidák-Holm-correction was used to correct for repeated comparisons.

Participants 1–12 took part in the online session first, while participants 13–24 took part in the in person session first. A possible cross-over effect was analyzed by comparing possible contrasts in differences between reported scores of the two training sessions. A possible cross-over (order) effect was assessed for each variable (RPE, RPD, sPDF, EES) by comparing, between the two order groups, the within-subject difference in scores between the online and in-person session (online minus in-person) using independent-samples t-tests. Resulting *p*-values were corrected for repeated comparisons using the Bonferroni-Šidák method.

### Qualitative data

The open-ended responses were analyzed using an inductive qualitative content analysis approach (28). Responses to the open-ended question were reviewed and coded into categories by three individual coders (TEJS, JIM & EH) working independently, after which the coders met to compare their coding, discuss discrepancies, and reach consensus, with the first author facilitating these discussions and resolving any remaining disagreements. Because coding was performed manually rather than logged in a shared digital format, formal inter-coder agreement statistics (e.g., Cohen's kappa) were not calculated; category assignment instead relied on discussion-based consensus among the three coders. Each response was coded for (1) overall preference and (2) categorization, with individual responses eligible for more than one category. All quotes for each category are presented in (Supplementary file 1).

## Results

### Affective responses

The participants scored the session pleasure lower during online the HIFT session compared with the in-person HIFT session (2.0 ± 2.2 vs. 3.1 ± 1.7, respectively, *p* = 0.02), as were the scores for exercise enjoyment (4.2 ± 1.2 vs. 5.1 ± 1.0, *p* = 0.004). Further, the participants reported less perceived effort during the online HIFT session than during in-person HIFT session (5.6 ± 1.7 vs. 6.7 ± 1.7, respectively, *p* = 0.02). There was no difference in perceived discomfort (4.4 ± 2.0 vs. 4.7 ± 1.9, *p* = 0.55). (See Figure 3 and Table 1 for details).

**Figure 3:**
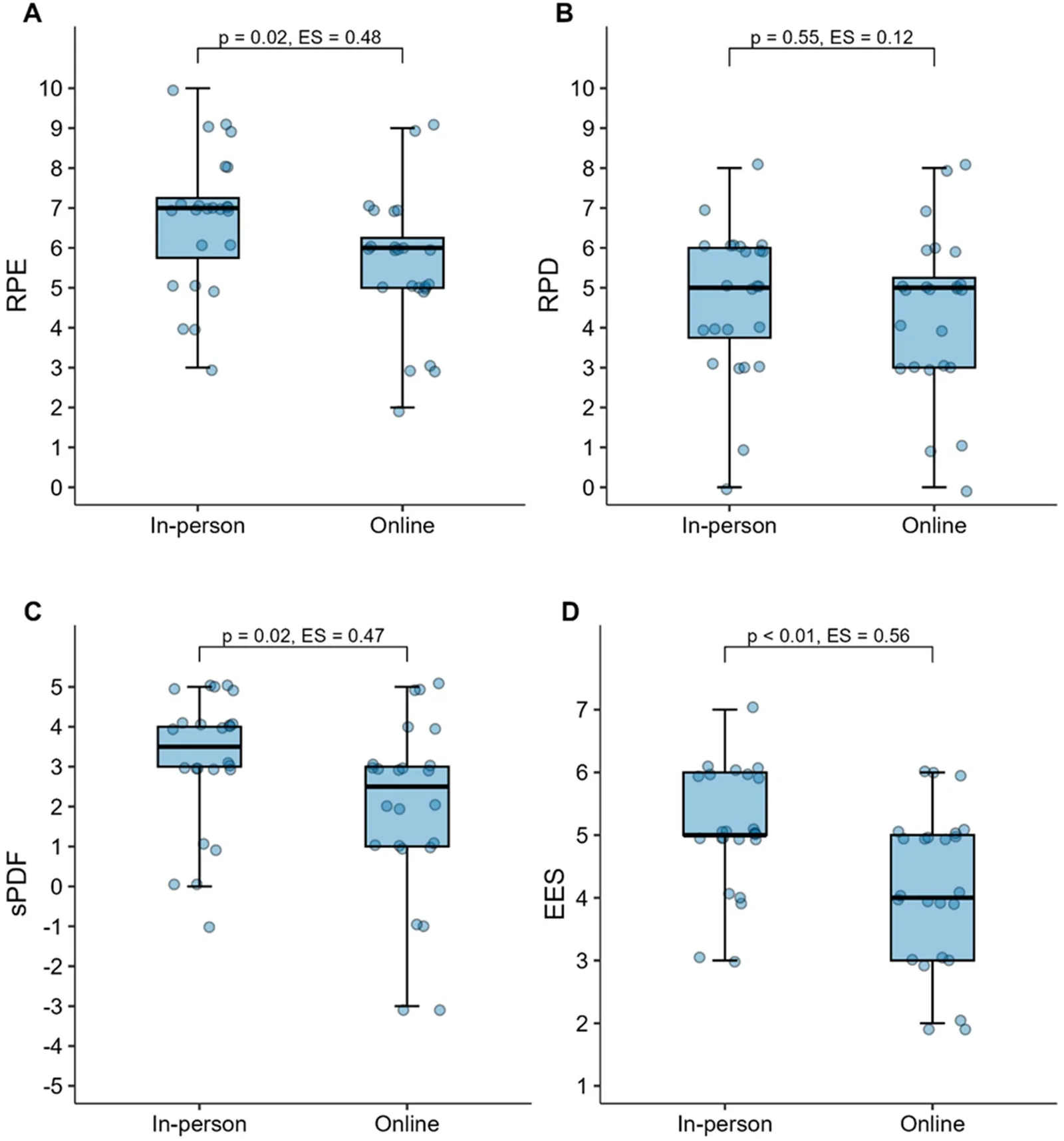
Affective responses per modality. Individual datapoints, median (central horizontal line), interquartile range (box). The vertical lines (whiskers) mark the limit 3 times the IQR below or above, respectively, the box. Individual values are also shown, and the dodge function in JASP has been used to separate point with similar values. **(A)**, rate of perceived effort (RPE); **(B)**, rate of perceived discomfort (RPD); **(C)**, session pleasure/displeasure feelings scale (sPDF); and **(D)**, exercise enjoyment scale (EES). ES = effect size. The scale on the y-axes show the whole range of possible values.

**Table 1:** Summary of answers to questionnaires .

Parameter	mean	SD	CI_0.95_ of difference	Median	Q_1_	Q_3_
RPE online	5.6	1.7	0.10 to 2.15	6	5	6.25
RPE in person	6.7	1.7		7	5.75	7.25
RPD online	4.4	2.0	−0.89 to 1.39	5	3	5.25
RPD in person	4.7	1.9		5	3.75	6
sPDF online	2.0	2.2	0.22 to 2.11	3	1	3
sPDF in person	3.1	1.7		4	3	4
EES online	4.2	1.2	0.32 to 1.51	4	3	5
EES in person	5.1	1.0		5	5	6

RPE, rates of perceived effort, RPD, rate of perceived discomfort, sPDF, session pleasure/displeasure feelings, and EES, scores on an exercise enjoyment scale. SD, standard deviation, CI_0.95_, 95% confidence interval, and Q_1_ and Q_3_ are the first and third quartiles, respectively.

Scores on the four affective responses were analyzed for possible correlations for the in-person and online sessions separately. Spearman's rank correlation analysis showed that for the online session almost all correlations were statistically significant (see Table 2, values below the diagonal)) excluding RPE-sPDF. For the in-person session, only RPE–sPDF and sPDF–EES scores correlated significantly (Table 2, values above the diagonal).

**Table 2:** Summary of mode-specific spearman's correlations between affective responses. .

Measurements	RPE	RPD	sPDF	EES
RPE		0.36	**0**.**61**	0.29
RPD	**0**.**62**		0.12	0.01
sPDF	0.50	**0**.**62**		**0**.**61**
EES	**0**.**57**	**0**.**65**	**0**.**56**	

Correlations for the in-person session are shown above and to the right of the diagonal, while values below and to the left of the diagonal are for the online session. Values being statistically significant after Bonferroni–Holm-corrections for repeated comparisons are boldfaced.

### Cross-over analyses

Participants 1–12 participated in the online session first and the in person session last. For participants 13–24 the order was exchanged. There was no systematic effect of the order of the training sessions (*P* ≥ 0.29 after Bonferroni-Šidák-correction for repeated comparisons). Combining two or more scores (e.g., pooling differences in RPE and RPD scores) did not give any statistically significant differences either.

### Content analysis

Twenty-two participants (92%) preferred in-person group exercise, while two participants (8%) selected “Group training via Zoom with online participation” as the preferred training modality. From the individual open-ended text answers, three main categories emerged.

#### Category 1: Motivation and performance

Responses related to motivation and performance were the most common across all the 22 participants who preferred the in-person modality. All of the 22 participant (100%) described increased motivation and effort, noting that it was easier to push themselves and work harder when attending in-person as a sort of social facilitation. As one participant explained, “*I managed to work better when I had people around me*” (#su7), while another noted that “*much more motivating, easier to push yourself with others around*” (#3).

#### Category 2: Social and relational

Twelve participants (55%) highlighted a sense of community and belonging as a reason for preferring in-person attendance, describing the social dimension as a valued part of the experience. One participant reflected that “*it becomes more than just training—a kind of social gathering*” (#2), and another said “you are social at the same time as you train” (#24). Eight participants (36%) mentioned instructor contact and feedback, noting that proximity to the instructor made it easier to follow instructions and receive cues during the session, as illustrated by one participant's comment that it was “*easier to catch instructions*” (#16) when training in person.

#### Category 3: Practical and logistical

Five participants (23%) mentioned better gym facilities, including music, space, and mirrors, as reasons for favoring in-person attendance, were one noted having “*more space and mirrors*” (#13). Five (23%) noted practical disadvantages of training at home, such as the risk of cutting corners, limited space, or consideration for housemates regarding noise. Four participants (18%) reported technical difficulties with Zoom as a drawback of the online format, describing problems such as “*technical problems, freezing and crackling, you can't follow what to do*” (#18). Notably, seven participants across all preference groups (29%) acknowledged that the online format could serve as a useful alternative when schedules did not allow for in-person attendance, with one noting that “*a Zoom session works well when the barrier to leave the house is high*” (#1).

Both participants who preferred the online session, cited convenience and time efficiency as their primary reason. One of them noted that the session, which required no equipment, was particularly well suited for a home environment, reflected in the comment that it was “*easier to set up the screen for an hour than to travel to the gym*” (#22).

## Discussion

The main finding of the present study was that the in-person HIFT session was acutely perceived as more pleasurable, enjoyable and effortful, compared with the online HIFT session with moderate to large effects (ES = 0.47–0.56). However, there was no difference in the perceived discomfort. The order of the two training sessions carried out did not affect any of the reported scores. Finally, when having to choose between the two sessions, twenty-two out of twenty-four preferred the in-person session.

The higher perceived in-person session pleasure and enjoyment, may be explained by the social component. It has been suggested that social identity from exercising in groups may lead to increased positive affections regarding the physical activity (29). Exercising together physically may increase the feeling of groupness and therefore increase the social dimension (30). This speculation is supported by the content analysis where 17 of the participants highlighted social and relational reasons (proximity to other participants and the instructor) for preferring the in-person session. This explanation aligns with the findings of previous studies, reporting the perception of groupness to be important for the perception of pleasure and enjoyment (30, 31). Our findings are also supported by a recent study comparing traditional and online sessions (13). The authors reported higher enjoyment scores for the traditional classes. In addition to the social factors, external factors such as the quality of the delivery of the online classes, was important for the perception of the classes (13). This could possibly explain the findings of the present study as five of the participants (23%) had practical or logistical arguments, such as music quality, technical difficulties, narrow space or disturbing others in the household for not preferring the online session. Furthermore, training related factors such as training experience (weekly training volume, HIFT, online) and physical fitness level could potential influence the perceived effort, enjoyment and preference. The generalization of findings should therefore be done with caution. Of importance, previous studies have often compared enjoyment during online and in-person training, but not conducted controlled designs (i.e., same training online and in-person) (13, 14). By including two independent variables (online vs. in-person and different fitness sessions), the interpretation of the findings are difficult. Furthermore, low-intensity fitness modalities like yoga may be a more viewable and preferred online option than HIFT (5), especially when the participants are middle-aged adults or patients with obesity (15, 16).

The acute perception of effort was perceived as higher in the in-person session compared with the online session. This may be explained by the social factor, as it has been reported that the feeling of groupness may explain higher motivation for increased effort in physical sessions (30). This argument is strengthened by our content analysis reporting that all the participants who preferred the in-person session, reported it as easier to push themselves when being in a more social setting. Of note, previous studies have not included effort or discomfort comparing online with in-person training (13, 14). The training related responses and physical adaptions are highly affected by intensity, therefore these outcomes provide a novelty of the present study. Notably, no objective measures were collected during the sessions, e.g., heart rate, number of repetitions, accelerometer-data etc. Consequently, we can`t exclude the possibility that the difference in RPE may be related to a difference actual work performed (repetitions performed, intensity in each repetitions etc.) and not online vs. in-person *per se*. Importantly, all participants had their cameras switched on during the digital session to ensure that all participated. Further, the information and motivation given by the instructor before and during the sessions were standardized to try to keep the settings as similar as possible.

In the present study, the in-person HIFT sessions led to both acute higher effort and more pleasure and enjoyment. This finding contradicts the findings of Maher et al. (31) where twenty-five adults participated in different types of in-person group training classes over a period of thirty weeks and subsequently reported their perception of each class. In general, the authors reported a trend that more intense classes were perceived as less intrinsic satisfying. The disparity between this and the present study may be a consequence of different populations and study design. In Maher et al. (31) longitudinal study, the participants were community-living adults with the majority being classified as overweight or obese. In the present acute study, the participants were sports students with group training experience who may be more accustomed to training and find intense sessions more motivating. This speculation is supported by our results showing a large correlation between effort and session pleasure (r = 0.50 and 0.61) and small to large between effort and enjoyment (r = 0.29 and 0.57). Furthermore, previous studies on similar populations have reported trained participants to perceive exertive sessions to be more pleasurable and preferred compared to less exertive sessions (22, 32).

There was no difference between the sessions in terms of perceived discomfort (ES = 0.12). This might appear somewhat surprising given the observed difference in effort and the fact that there was a positive moderate to large correlation (r = 0.36 and 0.62) between effort and discomfort. Also, previous research has shown that effort and discomfort are related (19). Importantly, these are two different parameters where perceived effort is linked to the degree of maximal capacity being used in the session, while perceived discomfort is related to a general feeling of pain/ discomfort. Consequently, the perceived discomfort score will be affected by not only the intensity but also other factors that would affect the sensory feedback of the activity, such as training volume, rest intervals etc (33). Hence, the similarity in discomfort could be explained by the fact that both the in-person and online session were similar in design and execution (i.e., same exercises, work intervals, rest intervals etc.).

There are some limitations that need to be addressed. First, no power sample analysis was conducted before starting the project, and a sample of convenience was included as we had no clear inferential goal (17). With small, heterogeneous samples, it's always a risk of conducting type II errors. Second, only physically active and healthy young adults (sports students) were recruited with limited data of the training realtered factors such as online training and HIFT experience, weekly distribution of strength and endurance training, physical fitness level. Thus, the findings cannot necessarily be generalized to other populations. Third and as mentioned, the outcomes only include the subjective experience of the in-person and online training sessions. Objective measurement such as work (e.g., number of repetitions etc.) or intensity (e.g., heart rate) could underline and confirm the findings from the perceived effort, perceived discomfort, session pleasure/displeasure and/or enjoyment. Furthermore, as no objective measures were included we cannot exclude the possibility of differences in work performed between the sessions (number of repetitions, intensity in each repetitions etc). Factors concerning the online delivery, such as each participant's home environment, internet connection and available space could influence enjoyment, effort and preference. Finally, the present study used a randomized crossover design, where only acute responses were investigated. Hence, longitudinal effects of the two training modalities cannot be addressed.

From a practical point of view, the finding of the present study implies that practitioners, trainers and gym facilities should focus on in-person group sessions, for sports students if the aim is higher acute effort, pleasure and enjoyment. Importantly, both sessions led to high levels of perceived effort, session pleasure and enjoyment and could therefore be good alternatives for practitioners based on their personal preferences. Furthermore, both participants who preferred the online session, argued this session to be more time efficient. This might imply that for populations with less time available, online group training is a more feasible alternative than for the population in the present study (sports students). The findings should not be generalized to beginners, older adults, less active adults, or individuals who may prefer private home-based training. Future studies should focus on the longitudinal effects of online vs. in-person sessions, examine other populations and include objective measurements.

## Conclusions

In conclusion, physical active sports students with prior group-training experience acutely perceived in-person group training more pleasurable, enjoyable and effortful with moderate to large effects, when compared to synchronously online group training. Furthermore, 92% of the participants (22 out of 24) preferred the in-person session if they had to choose only one of the sessions.

## Data Availability

The raw data supporting the conclusions of this article will be made available by the authors, without undue reservation.
