# Supplementary material for: Comparison of affective responses between an online and in-person high-intensity functional training session in sports students
Source: Front Sports Act Living. 2026 Sep 24;8:1916043. doi: 10.3389/fspor.2026.1916043 (PMC13646083; doi:10.3389/fspor.2026.1916043)
Supplement: Supplementary file 1 [file Datasheet1.pdf]

## Coding Overview

Open-ended responses. For categories 1 and 2 are of in-person respondents (n = 22); category 3 percentages are of all respondents (n = 24). Responses were collected in Norwegian; all quotes have been translated by the authors. Individual responses may be coded under more than one category.

| Category | n | % | Respondents and key phrases (translated from Norwegian) |
|---|---|---|---|
| **Overall preference (n = 24)** | | | |
| In-person | 22* | 92% | *two participants initially said they enjoyed both modalities equally, but the description after leaned heavily towards the in-person modality. So to make the analysis more concise and straightforward, these two were put in the "in-person" category. |
| Digital | 2 | 8% | |
| **Category 1: Motivation & Performance (n = 22 in-person)** | | | |
| *1a. Increased motivation and effort* | 22 | 100% | **#2:** *"it is easier to motivate yourself, push yourself harder"*<br>**#3:** *"much more motivating, easier to push yourself with other around"*<br>**#5:** "higher effort with people around then alone in the living room"<br>**#6:** *"more motivating because of the people around"*<br>**#7:** *"I managed to work better when I had people around me"*<br>**#8:** *"much easier to push yourself in a room with others"*<br>**#10:** *"more motivating then exercising alone"*<br>**#11:** *"motivated in a better way, give a little extra effort"*<br>**#12:** *"I performed more by being present"*<br>**#13:** *"easier to push yourself with the instructor in the same room"*<br>**#17:** *"more motivating to be in a gym where you can see the others"*<br>**#18:** *"easier to push a little extra"*<br>**#20:** *"motivation, performance and output"*<br>**#21:** *"easier to push yourself when together with others"*<br>**#23:** *"extra motivation from training with more people around"*<br>**#24:** *"most motivating to train together with others"*<br>**#5:** *"you perform a little more when others are present"*<br>**#9:** *"many people watch you, so you give extra energy even when exhausted"*<br>**#14:** *"push a little harder when others are watching"*<br>**#15:** *"you feel more pressure and the others around you push you"*<br>**#19:** *"you manage to perform a little better when training with others"*<br>**#11:** *"inspired by how others do it"* |
| **Category 2: Social & Relational (n = 22 in-person)** | | | |
| *2a. Sense of community and belonging* | 12 | 55% | **#2:** *"it becomes more than just training – a kind of social gathering"*<br>**#4:** *"much more present together with the other participants"* |

| | | | |
|---|---|---|---|
| | | | **#5:** *"also more social"*<br>**#6:** *"can socialize while training"*<br>**#8:** *"you feel like part of a community in the gym"*<br>**#10:** *"it becomes a social setting"*<br>**#11:** *"the feeling of social community, which is positive"*<br>**#12:** *"nice socially"*<br>**#13:** "fun to exercise with others"<br>**#15:** *"I got more of the group class feeling"*<br>**#16:** *"a different sense of belonging in the group when meeting physically"*<br>**#24:** *"you are social at the same time as you train"* |
| *2b. Instructor contact and feedback* | 8 | 36% | **#9:** *"the group instructor cheers you on during the session"*<br>**#11:** *"easier to take in feedback along the way"*<br>**#12:** *"better understanding of the exercises"*<br>**#13:** *"easier to push yourself with the instructor in the same room"*<br>**#16:** *"easier to catch instructions"*<br>**#18:** *"closer to the instructor"*<br>**#20:** *"motivated by instructor and participants; easier to catch instructions"*<br>**#21:** *"easier to receive instructions when there in person"* |
| **Category 3: Practical & Logistical (n = 24, all respondents)** | | | |
| *3a. Gym facilities and environment* | 5 | 23% | **#2:** *"the music experience is much stronger when you are in the gym"*<br>**#13:** *"more space and mirrors"*<br>**#14:** *"poor training environment in the room at home"*<br>**#19:** *"better facilities around you"*<br>**#20:** *"loud music"* |
| *3b. Technical difficulties with digital format* | 4 | 18% | **#7:** *"problems with the internet, kept dropping in and out of the Zoom room"*<br>**#16:** *"not dependent on whether the internet works"*<br>**#18:** *"technical problems, freezing and crackling, you can't follow what to do"*<br>**#20:** *"hard to catch instructions digitally"* |
| *3c. Disadvantages of training at home* | 5 | 23% | **#5:** *"easier to cut corners when others are not present"*<br>**#5:** *"not practical to train in the living room"*<br>**#9:** *"easier to skip exercises when at home"*<br>**#15:** *"had to consider housemates because of noise"*<br>**#15:** *"felt uncomfortable training at home"* |
| *3d. Digital format acknowledged as useful alternative* | 7 | 29% | **#1:** *"a Zoom session works well when the barrier to leave the house is high"*<br>**#9:** *"a good alternative for those who cannot get to the gym"*<br>**#10:** *"a good alternative if a new pandemic occurs"*<br>**#14:** *"Zoom was also good"*<br>**#19:** *"Zoom is a good alternative when everyday life is hectic"* |

| | | | |
|---|---|---|---|
| | | | **#22:** *"easier to set up the screen for an hour than to travel to the gym"*<br>**#22:** *"the exercises were best suited to doing at home"* |
